# How Do Honeybees Attract Nestmates Using Waggle Dances in Dark and Noisy Hives?

**DOI:** 10.1371/journal.pone.0019619

**Published:** 2011-05-16

**Authors:** Yuji Hasegawa, Hidetoshi Ikeno

**Affiliations:** 1 Honda Research Institute Japan Co. Ltd., Wako-shi, Saitama, Japan; 2 School of Human Science and Environment, University of Hyogo, Honmachi, Shinzaike, Himeji-shi, Japan; Alexander Flemming Biomedical Sciences Research Center, Greece

## Abstract

It is well known that honeybees share information related to food sources with nestmates using a dance language that is representative of symbolic communication among non-primates. Some honeybee species engage in visually apparent behavior, walking in a figure-eight pattern inside their dark hives. It has been suggested that sounds play an important role in this dance language, even though a variety of wing vibration sounds are produced by honeybee behaviors in hives. It has been shown that dances emit sounds primarily at about 250–300 Hz, which is in the same frequency range as honeybees' flight sounds. Thus the exact mechanism whereby honeybees attract nestmates using waggle dances in such a dark and noisy hive is as yet unclear. In this study, we used a flight simulator in which honeybees were attached to a torque meter in order to analyze the component of bees' orienting response caused only by sounds, and not by odor or by vibrations sensed by their legs. We showed using single sound localization that honeybees preferred sounds around 265 Hz. Furthermore, according to sound discrimination tests using sounds of the same frequency, honeybees preferred rhythmic sounds. Our results demonstrate that frequency and rhythmic components play a complementary role in localizing dance sounds. Dance sounds were presumably developed to share information in a dark and noisy environment.

## Introduction

It has been shown that honeybees use dance communication to share information related to colony maintenance [1]–[3]. Recruiters engage in a unique behavior called a waggle dance, which contains elements that encode the location of a food source and conveys this information to nearby followers. These followers use this information to collect food from the specified location. Although many honeybees engage in hive-related behavior unrelated to dance communication, follower bees are only able to search for dancers and communicate with them [4]–[7]. In order to orient themselves toward dancing bees, followers require the ability to detect features specific to dancing bees.

Some honeybee species (e.g., “dwarf bee” *Apis florea*, “rock bee” *Apis dorsata*) conduct dances on the upper outdoor surface of the hive, but others (e.g., “hive bee” *Apis mellifera, Apis cerana*) conduct dances in the hive. During their dances, honeybees periodically waggle their bodies from side to side with their wings. Members of the former species vigorously waggle their abdomens in the open while dancing and are visually conspicuous. By contrast, members of the latter species dance inside the hive where it is too dark to utilize visual features. Some signals that may assist hive dancer in communicating with followers have been suggested [1], [8], [9]. For example, various chemicals are produced and released by dancers to stimulate follower foraging. Floral odors are also thought to serve as cues that allow followers to locate the food source. However, it is still unclear whether followers are able to utilize odor cues to find dancers in hives, given that several different odors exist in the hive because of food storage and the activities of nestmates.

In dances conducted within a hive, a honeybee repeatedly runs in a particular direction along the comb while waggling its body from side to side [1], [2]. During the waggling run it also emits a burst of sound by buzzing its wings. The sounds consist of pulses, each pulse with a duration of approximately 20 msec and a carrier frequency of about 250–300 Hz [4], [5]. Previous studies have demonstrated that bees use Johnston organs, which sense sounds, to tune into this frequency [10]–[12]. One study found that after honeybees associated a 265-Hz tone stimulus with a reward following operant conditioning, they were able to localize the sound source and discriminate the frequency from a different frequency in a Y-maze [11]. However, dance communication is not the only hive-related behavior that includes wing vibrations. Other actions, for instance hive cooling, produce wing vibrations that emit sounds that are similar in frequency to dance sounds. In studies of comb vibrations, signal amplification by the phase-reversal phenomenon caused by repeated waggle runs was suggested to be effective in attracting dance followers in a noisy environment [13]. These vibration signals are sensed by proprioceptors in bees' legs that respond in the range of 200–1000 Hz [14].

**Figure 1:**
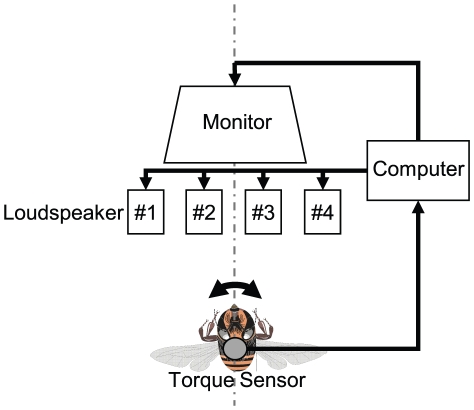
Experimental setup of sound source localization. A honeybee is tethered to a torque sensor that converts the bee's yaw torque into voltage. Based on this voltage, a computer controls the operating state of four loudspeakers and the position of a random dot pattern (horizontal [H] × vertical [V]  = 10×10 pixels) on a monitor. The loudspeakers and the monitor are located in front of the honeybee. The loudspeakers are placed at 60° (#1), 15° (#2), −15° (#3) and −60° (#4), respectively. The display size of the monitor is 640×480 pixels (H × V), which yields a 120×90 degree (H × V) view angle from the point of view of the tethered honeybee.

It is still unclear whether honeybees can use the ability to distinguish between the vibrations of waggle dances and those of other behaviors to find dancers in natural hives. A key question thus arises: How are dancers able to attract followers despite their noisy environment? Followers must detect these signals without being disturbed by wing vibrations secondary to other behaviors. In this study we used a flight simulator (Fig. 1) to investigate whether followers can discriminate dance sounds from wing vibrations arising from other behaviors. Honeybees were attached to a torque meter in order to analyze their orienting responses caused only by sounds, and not by odor or vibrations sensed with their legs. We then explored why the bees use rhythmic components of dance sounds.

## Results

### Experiment 1: Sound source localization toward a single sound source

**Figure 2:**
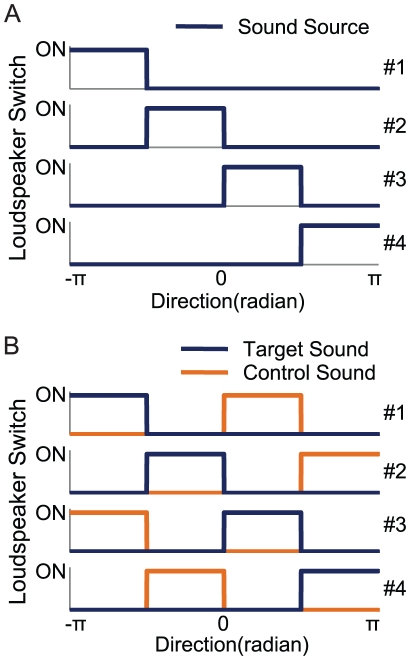
Control sequences between a honeybee's directions and the loudspeakers. The graphs demonstrate localization using either a single sound source (A) or two sound sources (B). The vertical axes show the state of each loudspeaker's switches (ON or OFF). The numbers on the right side of the graph indicate the loudspeaker number. The directions of each sound source were set negatively proportional to the voltage output of the torque meter attached to the honeybee's thorax. Whether a loudspeaker was turned on or off and which sound source it produced was decided by the directions of the sound source.

**Figure 3:**
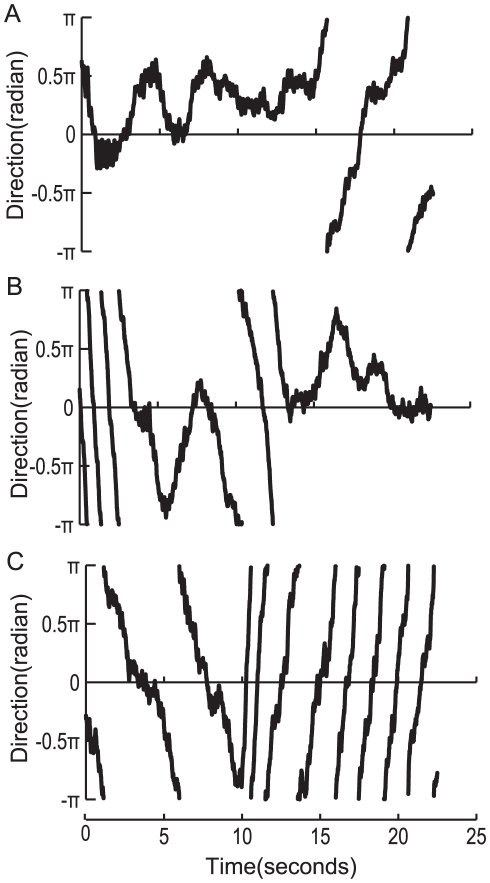
Temporal patterns of a honeybee's localization toward a single sound source. The time traces of a honeybee's orientation in response to flight sounds (A), dance sounds (B), and white noise (C) are shown. Each loudspeaker (#1, #2, #3, #4) produced a single sound source according to the honeybee's orientation. In dance or flight sound localization, honeybees tended to orient from −0.5π to 0.5π during their flights when the #2 or #3 loudspeakers produced dance or flight sounds. Honeybees did not orient in any particular direction when white noise was produced.

**Figure 4:**
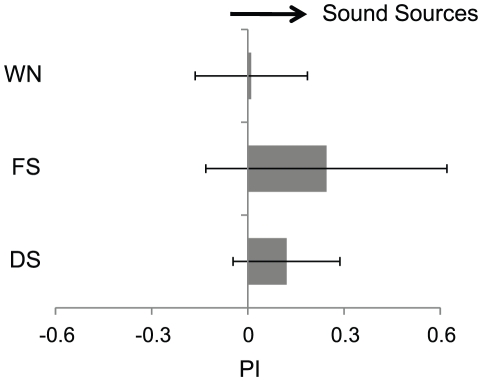
Honeybees' localization toward a single sound source. The left column indicates the types of sounds: dance sounds (DS), flight sounds (FS), and white noise (WN). Nine honeybees were tested in this experiment. The error bars indicate standard deviations. The grey areas show the mean performance indices (PI) for a 22-sec flight period. PI was calculated for these periods as a ratio of (t1−t2)/(t1+t2), where t1 indicates the duration during which the honeybee orients towards the current sound source and t2 indicates the duration during which the honeybee turns away from it. The starting position of a sound source was shifted randomly. Since PI's are 0.12±0.17 and 0.25±0.38 for dance (DS) and flight (FS) sounds, respectively, their non-zero hypotheses are significant with P<0.01. In contrast, since PI is 0.01±0.18 for white noise (WN), the non-zero hypothesis is not significant with P>0.05.

In order to verify the performance of sound source localization without conditioning in a flight simulator, honeybees localized single sound sources. We used dance sounds recorded in the natural hive, flight sounds of tethered honeybees, and white noise. The flight sounds were continuous sounds with a carrier frequency of about 250 Hz. Although dance sounds were constructed of pulse sounds differentiating them from flight sounds, the carrier frequency was 265 Hz, which is similar to flight sounds. White noise is a random signal with a flat power spectral density. Loudspeakers were located symmetrically, but not directly in front of the honeybees (Fig. 1). In addition, when honeybees flew leftward, their torque output increased (see Materials and Methods). A sound source shifted to the right loudspeaker. On the other hand, when bees flew rightward, a sound source shifted to the left loudspeaker (Fig. 2A). As a result, when honeybees oriented towards a sound source, their direction could be calculated by the output of their yaw torque oscillated at around 0 within the range from –π to π (tracking phase). In cases where honeybees did not orient toward a sound source, their direction tended not to show any oscillations (non-tracking phase). With flight sounds, the tracking phase occurred immediately following the trial and continued for about 15 seconds (Fig. 3A). On the other hand, with dance sounds the tracking phase began around 15 seconds after the trial began and continued until the end of the trial (Fig. 3B). Honeybees could localize the dance and flight sounds, and they preferred them to white noise. In single sound source localization for dance and flight sounds, two types of behavior appeared over the duration of the bees' flight (see Experiment 2). However, when exposed to white noise, honeybees did not fly in any particular direction so the tracking phase did not appear (Fig. 3C). To verify the time ratio between the time the bee is oriented a direction with the sound source and the time the bee is oriented a direction without it, we calculated the performance index (PI_s_), which was determined by (t_s_ − t_n_)/(t_s_ + t_n_), where t_s_ is the amount of time that a sound source was produced from loudspeaker #2 or #3 and t_n_ is the remaining time during which a sound source was produced from loudspeaker #1 or #4. The difference in PI_s_ among three groups (flight sounds, dance sounds and white noise) was not significant (Fig. 4; Steel−Dwass test; flight vs. dance: p = 0.99, flight vs. white noise: 0.27, dance vs. white noise: 0.25). However, within each group, PI_s_ showed that honeybees effectively localized dance sounds (PI_s_ = 0.12±0.17, p<0.01) and flight sounds (PI_s_ = 0.25±0.38, p<0.01), but not white noise (PI_s_ = 0.01±0.18). These results indicate that honeybees tend to prefer sounds with a carrier frequency of either 250 Hz or 265 Hz.

### Experiment 2: Sound source discrimination using different honeybee sounds

**Figure 5:**
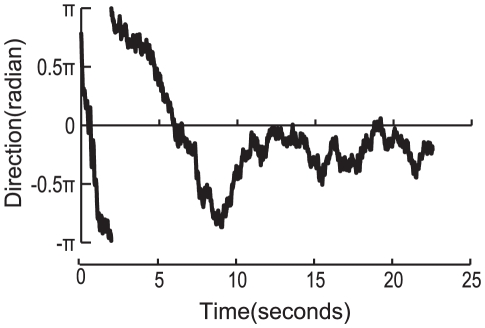
Temporal patterns of a honeybee's localization toward two sound sources. The time traces of a honeybee's orientation in response to flight and dance sounds are shown. Each loudspeaker (#1, #2, #3, #4) produced flight and dance sounds according to the honeybee's orientation. The time series variation was divided into two phases. The initial phase lasted for up to 10 seconds. During this phase the honeybee did not orient in any particular direction. After 10 seconds, it tended to orient from −0.5π to 0.5π, when the #2 or #3 loudspeakers produced dance sounds.

**Figure 6:**
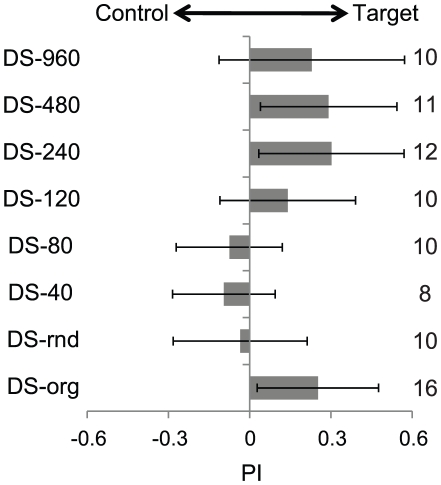
The effect of dance sound components on sound source discrimination. The numbers to the right of the grey bars indicate the number of honeybees tested. The error bars indicate standard deviations. The left column indicates the types of dance sounds: DS-org is the original dance sound, while DS-rnd and DS-n (n = 40–960, interval time [msec]) are synthetic dance sounds. The intervals in DS-rnd were randomly set between 40 msec and 960 msec. The mean interval length in DS-org was about 480 msec. The grey areas show the mean performance indices (PI) the 22-sec flight periods. PI is calculated for the periods as a ratio of (t1−t2)/(t1+t2), where t1 indicates the duration during which the honeybee orients towards the current dance sounds and t2 indicates the period during which the honeybee orients towards the flight sounds. Starting positions of sound sources were shifted randomly. Since PI's are 0.25±0.22, 0.30±0.27, 0.29±0.25, and 0.23±0.34 for dance sounds DS-org, DS-240, DS-480 and DS-960, respectively, their non-zero hypotheses are significant with P<0.01 (DS-org, DS-240 and DS-480) and P<0.05 (DS-960). On the other hand, since PI's are −0.03±0.25, −0.09±0.19 and −0.07±0.20 for dance sounds DS-rnd, DS-40, and DS-80, respectively, the non-zero hypothesis is not significant with p>0.05.

Two of the four loudspeakers simultaneously emitted sounds in order to investigate whether honeybees were capable of distinguishing between dance sounds and flight sounds. Sounds that honeybees oriented toward in the flight simulator were evaluated by the shifting of sound sources. When a tethered honeybee oriented toward a sound source, the source of this sound was then shifted to loudspeakers in front of the honeybee, and the other sound being produced at the same time was shifted toward peripheral loudspeakers (Fig. 2B). When both dance and flight sounds were produced, the behavior of honeybees could be divided into two phases. During the first 10 seconds, honeybees flew without orientating themselves to either sound, while after that, when it was the #3 loudspeaker that produced the dance sounds, they tended to orient from 0 to 0.5π (Fig. 5). It is possible that honeybees required time to search for each source, after which they were able to orient toward dance sounds. Calculation of PI (see Materials and Methods) to verify the time ratio between the time the bee is oriented a direction with the dance and flight sounds showed that in all cases, honeybees significantly oriented towards dance sounds (PI = 0.25±0.22, p<0.01) (Fig. 6, DS-org) rather than toward flight sounds. Thus, honeybees could distinguish between dance and flight sounds, and they preferred dance sounds.

### Experiment 3: Sound source discrimination using synthetic sounds

In the third experiment, we investigated which sound components were responsible for honeybees' identification of and ability to orient themselves toward dance sounds. Flight sounds are continuous sounds, whereas dance sounds are constructed of a rhythmic pulse signal with cyclic intervals. In order to test the effect of these differences, we presented several synthetic dance sounds at random or cyclic intervals with durations between 20 msec to 960 msec. Each sound presentation consisted of roughly 12 pulses, with each pulse having a duration of about 20 msec. In order to keep the sound power level consistent between presentations, each pulse was derived from a single set of pulses taken from original dance sounds. For synthetic sounds with a cyclic interval of 20 msec, we used repeated pulses rather than rhythmic sounds resembling original dance sounds. When their cyclic interval was 480 msec, their rhythm was closer to the original dance sounds. The difference among eight groups (Fig. 6, DS-org, rnd, 40–960) was significant (Fig. 6; ANOVA; F = 5.15, p<0.01). Tests with short intervals (40 msec: PI = −0.10±0.19, 80 msec: PI = −0.08±0.20) and random intervals (PI = −0.03±0.25) showed that honeybees oriented randomly towards flight and synthetic dance sounds or slightly preferred flight sounds (Fig. 6, DS-rnd, 40, 80). They were not able to distinguish between dance and flight sounds using only pulse sounds. However, they did prefer synthetic dance sounds at long cyclic intervals (more than 120 msec) (Fig. 6, DS-120, 240, 480, 960). Honeybees significantly preferred synthetic dance sounds with intervals of 240 msec (PI = 0.30±0.29), 480 msec (PI = 0.29±0.25) and 960 msec (PI = 0.23±0.34) (240 msec, 480 msec: p<0.01, 960 msec: p<0.05). In the case of 120-msec intervals (PI = 0.14±0.25), honeybees slightly preferred synthetic dance sounds (p>0.05). They were thus able to distinguish between dance and flight sounds using rhythmic components, and their preferred rhythm was very close to that of original dance sounds.

## Discussion

Our results showed that honeybees were able to localize sounds with carrier frequencies of both 250 Hz and 265 Hz and were not able to distinguish between flight and repeated pulse sounds produced by the waggle dances. The experiment using different rhythmic dance sounds showed that honeybees were able to detect rhythmic sounds within a certain frequency range. We suggest that dancers can attract followers even in their noisy environments using frequency and rhythmic components of dance sounds.

### Frequency components of dance sounds

Flight sounds are continuous tone stimuli, whereas dance sounds are characterized by temporal changes. Dancers generate repeated pulse sequences caused by wing vibrations and intervals. However, the frequencies produced by wing vibrations during waggle dances were similar to those produced while in flight (dance: 265 Hz, flight: 250 Hz). Honeybees' wing movement is generated by the dorsoventral and the dorsolongitudinal muscle groups, and the range of variability in wing beat frequency is small, about ±4% [15], [16]. In addition, previous studies showed that wing vibration frequencies remain almost the same even under different conditions [17]. The frequencies of both flight and dance sounds may be within the same range due to restrictions in neuromuscular mechanisms. A wide variety of sounds in the honeybee hive are caused by wing vibration. For example, continuous wing sounds are generated when honeybees are cooling down the temperature of the hive. In many unrelated situations, therefore, the frequency of a honeybee's wing vibrations may be similar to those observed during flight or dance.

Honeybees sense tone stimuli using Johnston organs, which are in the pedicel of their antennae [11]. Johnston organs are precisely tuned to detect frequencies of 250–300 Hz, which includes the wing beat frequency. They are also unable to distinguish between continuous and pulsed sounds [18]. In our experiments honeybees could orient themselves toward flight or dance sounds (Fig. 4) because the carrier frequencies of both repeated pulses and continuous sounds might be within the responsive range of Johnston organs.

Whilst our study showed that flying honeybees orient toward tone stimuli, the physiological mechanisms by which they do so are still unclear. Normally Johnston organs might react to airflow based on tone stimuli with the airflow caused by ego-motion. In our experiment, honeybees were attached to a torque sensor, so airflow caused by flying should have had little effect on their Johnston organs. Furthermore, we adjusted loudspeaker power so as to cause the Johnston organs of tethered bees to respond to the airflow of tone stimuli [18]. In keeping with the findings of most dance communication studies we assumed that honeybees can detect tone stimuli using their Johnston organs, but it is not yet known how airflow velocity attracts honeybees in natural hives. This issue may constitute an interesting research perspective for the future. We hypothesized that honeybees might be able to orient themselves toward sounds caused by wing vibrations produced by the behavior of nestmates as well as dance sounds. Therefore, although they can gather around nestmates using the frequency component of sounds, they need to sense other sound components to find a dancer that is providing information about food sources.

### Rhythmic components of dance sounds

Dance sounds have rhythmic components because dancers generate repeated pulse sequences caused by wing vibrations and intervals. The rhythm is mainly characterized by the ratio of the duration of the repeated pulse sequences to the interval. In hives, the duration of the waggling run increases monotonously with flight distance [3]. The interval duration might also increase with flight distance. As a result, there might be several rhythm variations in the waggle dances. Our experiment using different rhythmic dance sounds synthesized with different interval durations showed that honeybees were able to detect rhythmic sounds within a certain range (Fig. 6). They might be able to adapt to follow several types of dances that use different rhythms. Previous studies have suggested that honeybees sense sounds using Johnston organs. However, they are not able to distinguish between continuous and rhythmic sounds [18]. When honeybees decide whether the sounds they hear include rhythms related to waggle dances, they require a period of time to discern pulse sequences or intervals and to detect their durations. Our experiments demonstrated this time delay in bees' detection of rhythms. In the experiment on localization of dance sounds as well as that involving discriminating between dance and flight sounds, honeybees were able to detect dance sounds after flying in a direction not oriented toward any presented sound for about 10 seconds, it could detect dance sounds (Figs. 3B, 5). However, in the experiments on localization of flight sounds, honeybees could orient toward sounds in a short amount of time (Fig. 3A). We assumed that the rhythm of dance sounds must be processed in the nervous system at a higher level than that of the Johnston organs.

### Why do honeybees use the rhythmic components of dance sounds?

Members of the honeybee species used in this work normally conduct dances inside their hives, where it is too dark for them to make use of the visual features of waggling dances. Dancers emit one or more scents related to food sources because of nectar stored in their honey sacs or pollen attached to their legs or bodies after forging, so it is possible that followers utilize scent cues to detect waggle dancers. However, we believe that odor information cannot be a significant cue because several types of odors are given off by the honey, nectar, and pollen that are stored in the hive.

Comb vibrations have been studied as a directive and a long-range cue, which assists followers in detecting and localizing the dancer [13]. In previous experiments on comb vibrations measured during waggle dancing, dancing bees produced vibrations in two frequency ranges: 15 Hz and 200–300 Hz. Honeybees sense these vibrations using their subgenual organ, which is very insensitive to vibrations below 100 Hz but is quite sensitive to vibrations in the 200–1000 Hz range [14]. In natural hives, however, honeybees also face the difficulty of having to identify these signals within a noisy environment, such as they have to identify these signals within a noisy environment. No previous studies have shown that the subgenual organ can select specific features of waggle vibrations from among other comb vibrations. The organ responds to pure sine wave signals within the 200–1000 Hz range. Hive sounds are as noisy as comb vibrations, since there are several types of sounds caused by wing vibrations produced by the behavior of nestmates while maintaining the hive. In this study, however, we found that honeybees could discriminate between dance and flight sounds and they could select dance sounds with specific rhythmic components.

We assume that the specific rhythmic characteristics of dance sounds allow dancers to attract followers in the noisy hive environment. That is, workers located near the entrance of a hive are seeking sound sources with frequencies around 265 Hz. When they find these sources, they orient themselves toward them and wait for intervals in the sounds to determine whether or not they are dance sounds. If the repeated intervals are within the range appropriate for dance sounds, the workers detect them and may follow them. However, longer periods are needed to detect rhythm than frequency because of the time needed to discern duration and interval periods. Frequency and rhythmic characteristics are utilized in complementary fashion to find dance sounds in a noisy environment.

### How do honeybees acquire the ability to discriminate between different sounds?

In our flight simulator experiments, honeybees could orient to dance sounds without any conditioning. Previous experiments have suggested that the rate at which a honeybee follows a dancer increases after a trip in which the follower had failed to find food sources [19]. In our experiments, foragers were captured before they reached their food sources. Such failed foragers might be highly motivated to follow dancers, so they might spontaneously orient toward the cue of dance sounds in our experimental setup. Furthermore, it has been shown that the courtship songs of some insects, for example crickets or Drosophila, normally have their own inherited rhythm [20]–[22]. Females are attracted to rhythms of their own species as a result of instinct rather than learned behavior. Similarly, dance sounds contain a specific rhythmic component, so we assumed that bees might respond to them instinctively.

Among honeybee species, dwarf bees, *Apis florae*, conduct dance communication on a single comb in the open, while rock bees, *Apis laboriosa*, always dance silently [23], [24]. It has therefore been suggested that these bees might be able to utilize visual information conveyed by their dance behavior [3]. Hive bees, *Apis mellifera*, however, conduct dances with rhythmic sounds in a dark hive. Studies of morphological and molecular characteristics have suggested that dwarf bees and rock bees diverged from hive bees such as the Western honeybee [25], [26]. The ability to detect the rhythmic components of sound might have evolved after this divergence.

### Conclusion

Our study suggests that honeybees conduct dances that produce rhythmic sounds to attract followers in a dark and noisy hive. The utilization of rhythmic components presumably evolved to allow bees to determine whether a target with information or the information itself could be detected in a dynamic environment. In the future it should be possible to study mechanisms of dance communication, especially the configuration of dance language, by tracking bees' behavior after they are stimulated by various synthetic dance sounds.

## Materials and Methods

### Procedure

The honeybees (*Apis mellifera*) used throughout this study were randomly collected near a hive entrance on sunny days between March and August 2007 at the University of Hyogo (Himeji City, Hyogo, Japan). Captured bees were immobilized by cooling them briefly, and a small piece of iron plate (size: 1 mm ×2 mm, thickness: 0.02 mm) was brazed with beeswax on their thorax. A torque meter was attached to the plate in order to monitor the bees' movement. The voltage output, v(t), of the torque meter was low-pass filtered and was recorded every 5 msec. To exclude head movements during the experiments, a small drop of beeswax was positioned between each bee's head and thorax. To analyze honeybees'orienting response caused by only sounds, but not by odor or vibrations sensed by its legs, we constructed a flight simulator [27], [28] (Fig. 1) in which a honeybee was attached to a torque meter (Suzuko SH-002S) with its head and thorax fixed, allowed it to control its yaw torque to locate sound sources. Four loudspeakers (Onkyo GX-77M) and one high-speed CRT display (Iiyama HM903DA) were located in front of a tethered honeybee. The loudspeaker positions were at −60° (#1), −15° (#2), 15° (#3) and 60° (#4). At any given time, only one or two of the four loudspeakers emitted sound. The virtual angular positions, p(t), were made negatively proportional to the voltage output of the torque meter:
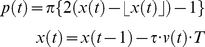
(1)where τ is the constant coefficient to convert torques to angular velocities, and T is the sampling time. This closed-loop mode allowed the stationary bees to control the horizontal rotation of the sound sources. The switching states, S_n_(t), of the four loudspeakers were established according to the virtual angular positions:
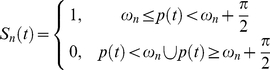
(2)where n is the loudspeaker number (#1, #2, #3, #4) and ω_n_ are constant values corresponding to each loudspeaker. When the switching states are S_n_(t)  = 1, the loudspeaker n produces the sound source. With a single sound source, ω_n_(n = 1, 2, 3, 4) are −π, −0.5π, 0 and 0.5π, respectively (Fig. 2A). Otherwise, with two sound sources, which include target sounds (e.g., dance sounds) and control sounds (e.g., flight sounds), ω_n_(n = 1, 2, 3, 4) for target sounds are −π, −0.5π, 0 and 0.5π, respectively, and ω_n_(n = 1, 2, 3, 4) for control sounds are 0, 0.5π, −π and −0.5π, respectively (Fig. 2B). Flying bees were able to stabilize their flight direction using visual stimuli, so a random dot pattern (horizontal [H] × vertical [V]  = 400×400 pixels) was associated with a sound source and the positions of patterns on the CRT moved in synchronization with the change of the position (p(t)) of sound sources. To prevent visual orientation and discrimination of patterns associated with sound sources, we used a single set of random dot patterns (dot size: 10×10 pixels, Michelson contrast: m = 0.99). Each random pattern moved horizontally across the monitor with the central position of each pattern shown along the horizontal axes. The patterns were positioned equal distances apart (600 pixels). Due to the limited size of the monitor (H × V  = 640×480), an entire pattern could not be fully displayed. According to the honeybee's yaw torque, the position of sound sources synchronized with the visual pattern shift.

### Sound sources

Dance sounds were recorded in the natural hive. Those sounds were produce by a single bee walking in a figure-eight pattern in its hive. As comparison sounds, flight sounds and white noise were used. Flight sounds were recorded from a tethered honeybee flying in the flight simulator (see Fig. 1). White noise, which is a random signal with a flat power spectral density, was synthesized by Adobe Audition (Adobe Systems Incorporated). The flight sounds were continuous sounds with a carrier frequency of about 250 Hz. Dance sounds were constructed as interrupted sounds with a frequency of about 265 Hz, differentiating them from flight sounds. That is, the dance sounds consisted of a rhythmic pulse signal with cyclic intervals. In order to test the effect of these differences, we prepared several synthetic dance sounds at random or cyclic intervals with durations between 20 msec and 960 msec. Each sound presentation consisted of roughly 12 pulses, each pulse with a duration of about 20 msec. In order to keep the sound power level consistent between presentations, each pulse was derived from a single set of pulses taken from original dance sounds. For synthetic sounds with a cyclic interval of 20 msec, we used repeated pulses rather than rhythmic sounds resembling original dance sounds. When the cyclic interval was 480 msec, the rhythm was closer to that of original dance sounds. Airflow velocities measured at tethered honeybees from each loudspeaker were about 0.3 mm/sec (measured by a hot wire probe: Microflown PU-probe).

### Experiment 1 (Sound source localization with a single sound)

We investigated whether honeybees could orient a sound source and what type of sound sources would be oriented. To establish a condition where the honeybee was quiet, it was attached to a torque meter and held a piece of paper. During the honeybee's flight after removing the paper from its legs, a single sound source was produced for 22 seconds from one of the four loudspeakers. Due to the fact that the legs of the tethered honeybees were hanging in the air, they could use their antennae to detect sounds but could not use their legs to detect vibrations. Sound source directions were shifted by alternating which of the four loudspeakers produced the sound, according to the torque caused by the honeybee's flight (Fig. 2A). Each time a sound source was produced by a loudspeaker. Performance index (PI_s_) was calculated as PI_s_  =  (t_s_ − t_n_)/(t_s_ + t_n_), where t_s_ is the fraction of time during which a sound source was produced from loudspeaker #2 or #3 and t_n_ was the remaining time during which a sound source was produced from loudspeaker #1 or #4. Although most honeybees continued flying during the trial while the sound was produced, some of them aborted their flights in the middle of a trial and thus their data were not included. Sound sources were produced by a loudspeaker from a random angular position at the beginning of each 2-min interval. Three types of sound sources (dance sounds, flight sounds, and white noise) were tested for each honeybee. The sound source sequences were played randomly.

### Experiments 2 and 3 (Sound source discrimination)

We investigated whether honeybees could discriminate between two sounds. During the honeybees' flights (see Experiment 1), two different sound sources were produced for 22 seconds from two of the four loudspeakers, respectively. When a sound source was produced from the central loudspeakers (#2 or #3), another sound source was shifted to the peripheral speakers (#1 or #4). Furthermore, in order to separate two sounds clearly, two sound sources were produced alternately from four loudspeakers. For example, when a sound source was produced from loudspeaker #1, another one was produced from loudspeaker #3. On the other hand, when a sound source originated from loudspeaker #2, another one came from loudspeaker #4 (Fig. 2B). In Experiment 2, we investigated whether honeybees could discriminate between dance sounds and flight sounds. In Experiment 3, we investigated whether honeybees could discriminate between synthetic dance sounds and flight sounds. Performance index (PI_d_) was calculated as PI_d_  =  (t_d_ – t_f_)/(t_d_ + t_f_), where t_d_ is the fraction of time during which dance sounds or synthetic dance sounds were produced from loudspeaker #2 or #3 and t_f_ is the remaining time during which flight sounds were produced from loudspeaker #2 or #3. The sound source was produced by a loudspeaker from a random angular position at the beginning of each 2-min interval. In Experiment 3, eight kinds of sound sources were tested as synthetic dance sounds for each honeybee. The sound source sequences were played randomly.

### Statistics

In all cases, we checked for normality using the Kolmogorov−Smirnov test. We also checked for equality of variance in the performance of groups within each experiment using the Levene test. In the case of a single sound source localization experiment, the Levene test was positive (p<0.05), so the Steel-Dwass test was used instead. On the other hand, in the case of sound source discrimination experiments, the Levene test's P value was not positive (p>0.05), so a one-factorial ANOVA was used. For each individual bee, we calculated the percentage of times a target sound source was chosen per test (i.e., a single value per bee). Performance in a given test was therefore assessed using a sample of such values. This situation allowed a one-sample approach in which our null hypothesis was that the percentage of times a target sound source was chosen in the test considered was not different from the theoretical value of 0. Such a hypothesis was evaluated by means of a one-sample t-test. In all cases the alpha level was 0.05.
